# Remote ischemic conditioning prevents ischemic cerebrovascular events in children with moyamoya disease: a randomized controlled trial

**DOI:** 10.1007/s12519-024-00824-z

**Published:** 2024-07-01

**Authors:** Shuang-Feng Huang, Jia-Li Xu, Chang-Hong Ren, Nathan Sim, Cong Han, Yi-Qin Han, Wen-Bo Zhao, Yu-Chuan Ding, Xun-Ming Ji, Si-Jie Li

**Affiliations:** 1 Department of Neurology, Xuanwu Hospital, Capital Medical University, No 45, Changchun Street, Xicheng District, Beijing, 100053 China; 2 Department of Rehabilitation Medicine, Beijing Shijitan Hospital Affiliated to Capital Medical University, Beijing, China; 3 Beijing Institute of Brain Disorders, Capital Medical University, No.10, Xitoutiao, You’anmenwai, Fengtai District, Beijing, 100053 China; 4 Beijing Key Laboratory of Hypoxic Conditioning Translational Medicine, Xuanwu Hospital, Capital Medical University, Beijing, China; 5 Department of Neurosurgery, Wayne State University, Detroit, MI USA; 6 Department of Neurosurgery, The Fifth Medical Centre, Chinese PLA General Hospital, Beijing, China; 7 Department of Emergency, Xuanwu Hospital, Capital Medical University, Beijing, China

**Keywords:** Asymptomatic moyamoya disease, Children, Remote ischemic conditioning, Stroke

## Abstract

**Background:**

Moyamoya disease (MMD) is a significant cause of childhood stroke and transient ischemic attacks (TIAs). This study aimed to assess the safety and efficacy of remote ischemic conditioning (RIC) in children with MMD.

**Methods:**

In a single-center pilot study, 46 MMD patients aged 4 to 14 years, with no history of reconstructive surgery, were randomly assigned to receive either RIC or sham RIC treatment twice daily for a year. The primary outcome measured was the cumulative incidence of major adverse cerebrovascular events (MACEs). Secondary outcomes included ischemic stroke, recurrent TIA, hemorrhagic stroke, revascularization rates, and clinical improvement assessed using the patient global impression of change (PGIC) scale during follow-up. RIC-related adverse events were also recorded, and cerebral hemodynamics were evaluated using transcranial Doppler.

**Results:**

All 46 patients completed the final follow-up (23 each in the RIC and sham RIC groups). No severe adverse events associated with RIC were observed. Kaplan–Meier analysis indicated a significant reduction in MACEs frequency after RIC treatment [log-rank test (Mantel–Cox), *P* = 0.021]. At 3-year follow-up, two (4.35%) patients had an ischemic stroke, four (8.70%) experienced TIAs, and two (4.35%) underwent revascularization as the qualifying MACEs. The clinical improvement rate in the RIC group was higher than the sham RIC group on the PGIC scale (65.2% vs. 26.1%, *P* < 0.01). No statistical difference in cerebral hemodynamics post-treatment was observed.

**Conclusions:**

RIC is a safe and effective adjunct therapy for asymptomatic children with MMD. This was largely due to the reduced incidence of ischemic cerebrovascular events.

**Graphical abstract:**

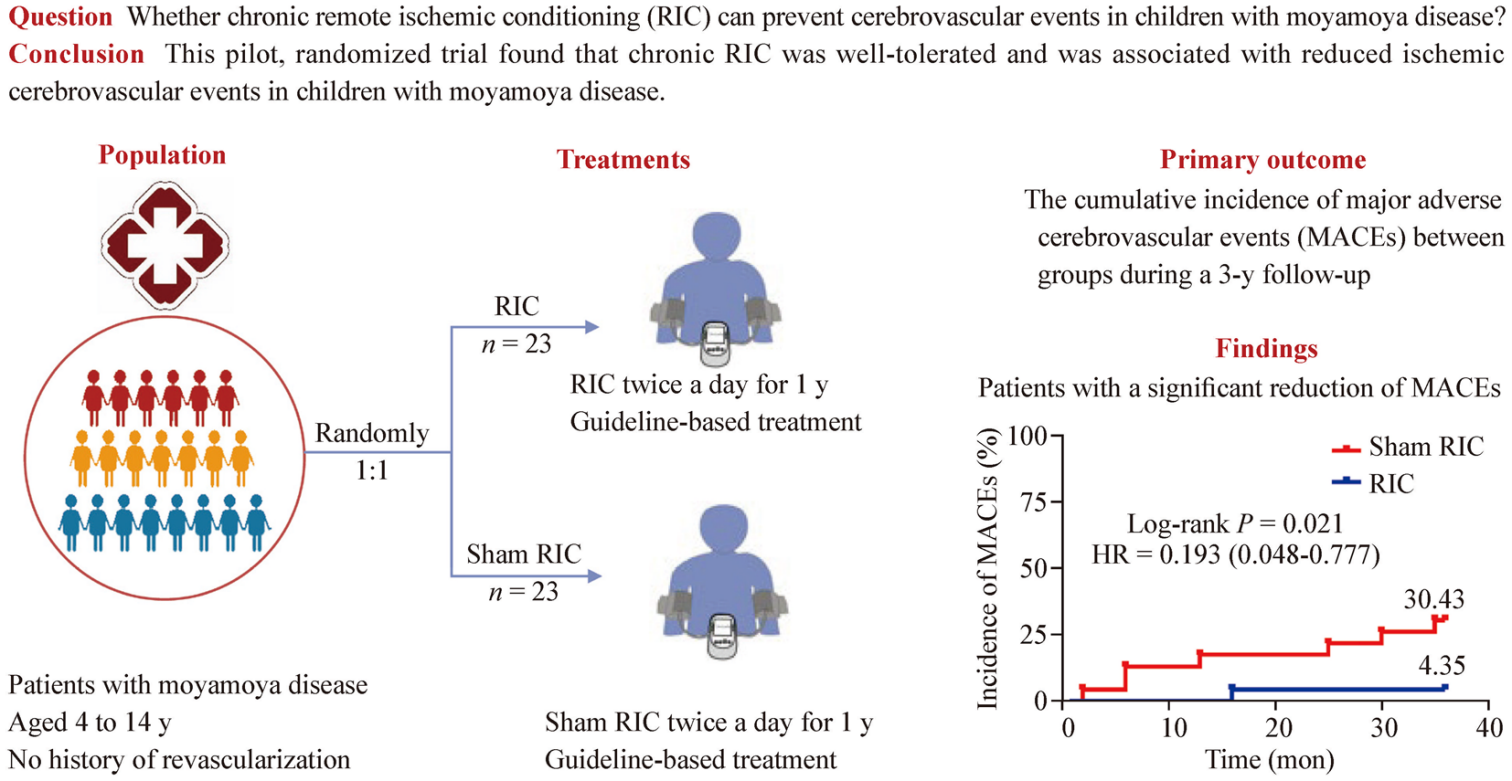

## Introduction

Moyamoya disease (MMD) is a chronic cerebrovascular disorder characterized by progressive stenosis or occlusion at the internal carotid artery end and abnormal net-like vessels at the brain base [1]. MMD carries a lifelong risk of stroke, with the highest onset in children aged 5–9 years, along with another small peak at 10–14 years [2, 3]. MMD frequently causes cerebrovascular events throughout the disease course in both children and adolescents but may also manifest with multiple and non-cerebrovascular clinical phenotypes, including headache (isolated or associated with other manifestations), seizures, movement disorder, and cognitive impairment [4, 5].

The mechanism of MMD has not yet been fully elucidated, and no curative treatment exists [6]. The standard of care for stroke prevention in children with symptomatic MMD is revascularization surgery [7, 8]. However, the treatment of children with asymptomatic MMD using revascularization surgery remains controversial. Asymptomatic MMD is not a silent disorder and may still cause ischemic or hemorrhagic strokes. Moreover, pediatric MMD tends to progress faster, and recovery after cerebral infarction is typically poorer compared to adult MMD patients [6]. Therefore, decreasing cerebrovascular events is a major factor affecting clinical outcomes in asymptomatic children with MMD without surgical revascularization.

Remote ischemic conditioning (RIC), comprising repeated episodes of transient limb ischemia and reperfusion, is a powerful brain protection intervention [9]. The neuroprotective mechanism of RIC involves the induction of angiogenesis and collateral formation, enhancement of collateral circulation, and protection of neurovascular units in the brains of ischemic animals, thus improving cerebral blood flow [9–11]. In addition, RIC can enhance tolerance to ischemia–reperfusion injury by reducing the duration and degree of hyperperfusion, modulating the inflammatory milieu of ischemic areas of the brain, and altering the immunological response to the ischemia [12]. Recently, the application of RIC for the treatment of MMD has been reported [13, 14]. One single-arm open-label study showed that RIC is a promising noninvasive technique for ischemic MMD control, alleviating symptoms, and minimizing stroke recurrence [13]. Another placebo-randomized controlled trial of 34 adult patients with MMD revealed that daily RIC could increase cerebral blood flow and slow the arterial progression of steno-occlusive lesions [14]. These studies have mainly focused on RIC as an adjuvant treatment for adult patients with MMD [13, 14]; however, the safety and effectiveness of RIC in children with MMD remain unknown. The clinical features of patients with MMD differ depending on their age, with children typically experiencing transient ischemic attacks (TIAs) and adults presenting with cerebral strokes [3]. This leads to stronger collateral circulation in children, which may enhance the neuroprotective effects of RIC [15]. Research investigating whether RIC can prevent neurological complications in children with MMD undergoing revascularization is underway (NCT 03546309) [16]. Therefore, we conducted a randomized controlled study to explore whether 1-year RIC treatment is safe and effective in asymptomatic pediatric patients with MMD without a history of reconstructive surgery.

## Methods

### Study design and participants

This was a single-center, randomized, double-blind, sham-controlled trial conducted at Xuanwu Hospital, Capital Medical University. The trial was registered at www.clinicaltrials.gov (unique identifier: NCT03821181), approved by the Ethics Committee of Xuanwu Hospital of Capital Medical University, and conducted in compliance with the ethical principles of the Helsinki Declaration. All participants or their legally authorized representatives provided informed consent before enrollment.

The inclusion criteria were: (1) aged 4 to 14 years; (2) undergoing digital subtraction angiography (DSA) and meeting the MMD diagnostic criteria recommended by the Research Committee of the Ministry of Health and Welfare of Japan [17]; (3) patients with no history of TIA, ischemic or hemorrhagic stroke, or involuntary movements attributable to MMD, which may involve nonspecific symptoms such as headaches, dizziness, cognitive decline, and emotional changes [18, 19]; (4) cerebral vascular reserve detected by single-photon emission computed tomography showing no severe impairments; (5) no neurologic deficits on physical examination; and (6) informed consent provided by a legal representative.

The exclusion criteria were: (1) previous cerebral revascularization; (2) secondary moyamoya phenomenon caused by sickle cell disease, neurofibromatosis type 1, cranial therapeutic irradiation, Down syndrome, congenital cardiac anomaly, renal artery stenosis, giant cervicofacial hemangiomas, and hyperthyroidism; (3) mental disorders that could mimic nonspecific moyamoya symptoms, such as schizophrenia, anxiety, mood disorder, bipolar disorder, and psychoactive substance abuse; (4) unlikely to be available for follow-up over 1 year; (5) severe hepatic or renal dysfunction; and (6) any of the following cardiac diseases: mitral and/or aortic stenosis, patent foramen ovale, bacterial endocarditis, or any other cardiovascular condition interfering with participation.

### Randomization and masking

Patients diagnosed with MMD by DSA were recruited, and baseline assessments were performed. Patients were then randomly assigned to the RIC or sham RIC group at a ratio of 1:1. Randomization was performed using opaque and sealed envelopes that contained the group allocation. The randomization numbers were computer-generated with a block size of six. An investigator who was not involved in the study design or data analysis opened the envelopes to the participants. All the participants, investigators, and examiners were blinded to the treatment assignments.

### Interventions

All enrolled patients underwent risk factor management and lifestyle guidance, including antiplatelet therapy, appropriate blood pressure control, avoiding hot meals (noodles, soup, etc.), strenuous exercise, playing wind instruments (such as flutes), and blowing balloons [20]. In addition, all participants were asked to complete either the RIC or sham RIC treatment twice daily for one year. The RIC protocol involved five cycles of bilateral upper arms with 5-minute inflation to 50 mmHg above systolic blood pressure alternating with 5-minute deflation using an automated device, whereas the sham RIC protocol involved five cycles of bilateral upper arms with 5-minute inflation to 30 mmHg alternating with 5-minute deflation. After completing the one-year RIC or sham RIC treatment, the participants were not permitted to continue the intervention.

An electronic autocontrol device (Xuanyitong, Beijing Renqiao, China) was used for the RIC and sham RIC procedures. Electronic devices were equipped with subscriber identity module cards containing patient-specific identification numbers and RIC implementation dates, which were linked to a background monitoring platform. To ensure RIC compliance, the background monitoring platform automatically reminded the patients if treatment had become interrupted for three consecutive days. The investigators were alerted when the RIC was interrupted for four consecutive days. This information was documented on a background monitoring platform. A “grace” period of one session of RIC was assessed to be approximately four days [21]. Compliance of one month was considered substandard if a patient interrupted RIC or sham RIC treatment for four consecutive days of the month.

### Follow-up

After the intervention began, clinical events were followed up for three years. All enrolled participants were required to undergo monthly follow-ups, or when necessary during the first year, and every two months, or when necessary, thereafter. During these visits, the patients were questioned about the occurrence of stroke or TIAs, revascularization surgery, RIC, current medications, management of cerebrovascular disease risk factors, and any other discomfort. The patient global impression of change (PGIC) assessment was performed both at the beginning and the end of the intervention. This information is recorded in the database.

### Outcome assessment

#### Efficacy

The primary outcome of this study was the cumulative incidence of major adverse cerebrovascular events (MACEs). MACEs were defined as the occurrence of stroke, recurrent transient ischemic attacks (≥ 2 attacks), or need for revascularization surgery as determined by the treating team. If participants experienced a stroke, recurrent TIAs, or revascularization surgery, any of these events were considered as MACE. Ischemic stroke occurrence refers to a new cerebral infarct lesion detected by diffusion-weighted imaging (DWI) or fluid-attenuated inversion recovery (FLAIR) weighted sequencing. Hemorrhagic stroke was diagnosed when DWI- or FLAIR-weighted sequences showed new hemorrhagic or high-density lesions on computed tomography (CT). TIA events were defined as neurological symptoms and signs that resolved spontaneously within 24 hours after onset, without evidence of infarction on magnetic resonance imaging (MRI) or CT. The decision for revascularization was made by neurosurgeons with experience in vascular reconstruction techniques. This decision was based on comprehensive evaluations of the patient’s condition and the willingness of the patient’s guardian to operate.

Secondary outcomes included the rates of ischemic stroke, recurrent TIAs, hemorrhagic stroke, revascularization surgery, and improvement in PGIC scales at follow-up assessment. The PGIC scale was used to assess the occurrence or progression of nonspecific symptoms and cerebrovascular events associated with MMD. The PGIC consists of a 5-point scale (1 = significant reduction or elimination of symptoms, 2 = mild attenuation, 3 = no change, 4 = mild deterioration, and 5 = significant deterioration). A significant reduction or elimination of symptoms was regarded as clinical improvement. Changes in overall symptomatology were recorded at outpatient follow-up.

#### Safety

Safety measures in this study were assessed based on the presence of upper limb injury, including local edema, petechiae, ecchymosis, skin lesions, and any adverse events related to RIC. These were assessed by two independent examiners who were blinded to the treatment assignments. Any differences in these assessments were resolved by attempting to reach an agreement between the examiners. If no consensus was reached, a third examiner performed the final assessment.

#### Cerebral hemodynamics evaluation

Transcranial Doppler (TCD) is a useful tool for evaluating and monitoring patients, as well as detecting unstable diseases [22, 23]. It is a noninvasive, low-risk, bedside technique that enables real-time characterization of cerebral hemodynamics. However, there is no exact numerical method to judge the instability of cerebral hemodynamics, given the relative rarity of cerebrovascular disorders in children and the age-related changes in cerebral hemodynamics.

This study evaluated patients using baseline Suzuki stages on cerebral angiography and TCD parameters, based on previous TCD research findings in pediatric patients [23, 24]. A prospective study showed that the mean cerebral blood flow velocity of the middle cerebral artery (MCA) by TCD in patients with MMD ≤ 21 years old was 159 cm/s [23]. In this study, we statistically analyzed the MCA with a baseline mean velocity ≥ 159 cm/s. All tests were conducted using a Hitachi Aloka Arietta 70 (2.0 MHz transducer) ultrasound machine. Measurements were performed only once for each vessel because pediatric patients could not tolerate prolonged examinations, including peak systolic velocity (PSV), end-diastolic velocity (EDV), and mean velocity (MV).

### Statistical analysis

This was a pilot randomized controlled study. No parameters were used to estimate the sample size. However, in a pilot study, Hertzog et al. showed that 10–20 participants per group were adequate to assess feasibility [25]. Twenty patients per group were, therefore, planned, and 46 patients were eventually selected for this study. To compare the characteristics of the RIC group with the control group at baseline and follow-up, continuous variables were analyzed using the independent Student’s *t *test or the Mann–Whitney *U* test and expressed as mean ± standard deviation or medians [interquartile ranges (IQRs)]. Categorical variables were analyzed using the *χ*^2^ test or Fisher’s exact test and shown in proportions. The cumulative incidence of MACEs for time-to-event variables was obtained using Kaplan–Meier estimates. All data were analyzed using SPSS (version 24.0; IBM Inc.), with the significance level set at *P* < 0.05. Kaplan–Meier curves were constructed using Prism (version 8.02; GraphPad Software Inc.).

## Results

**Fig. 1:**
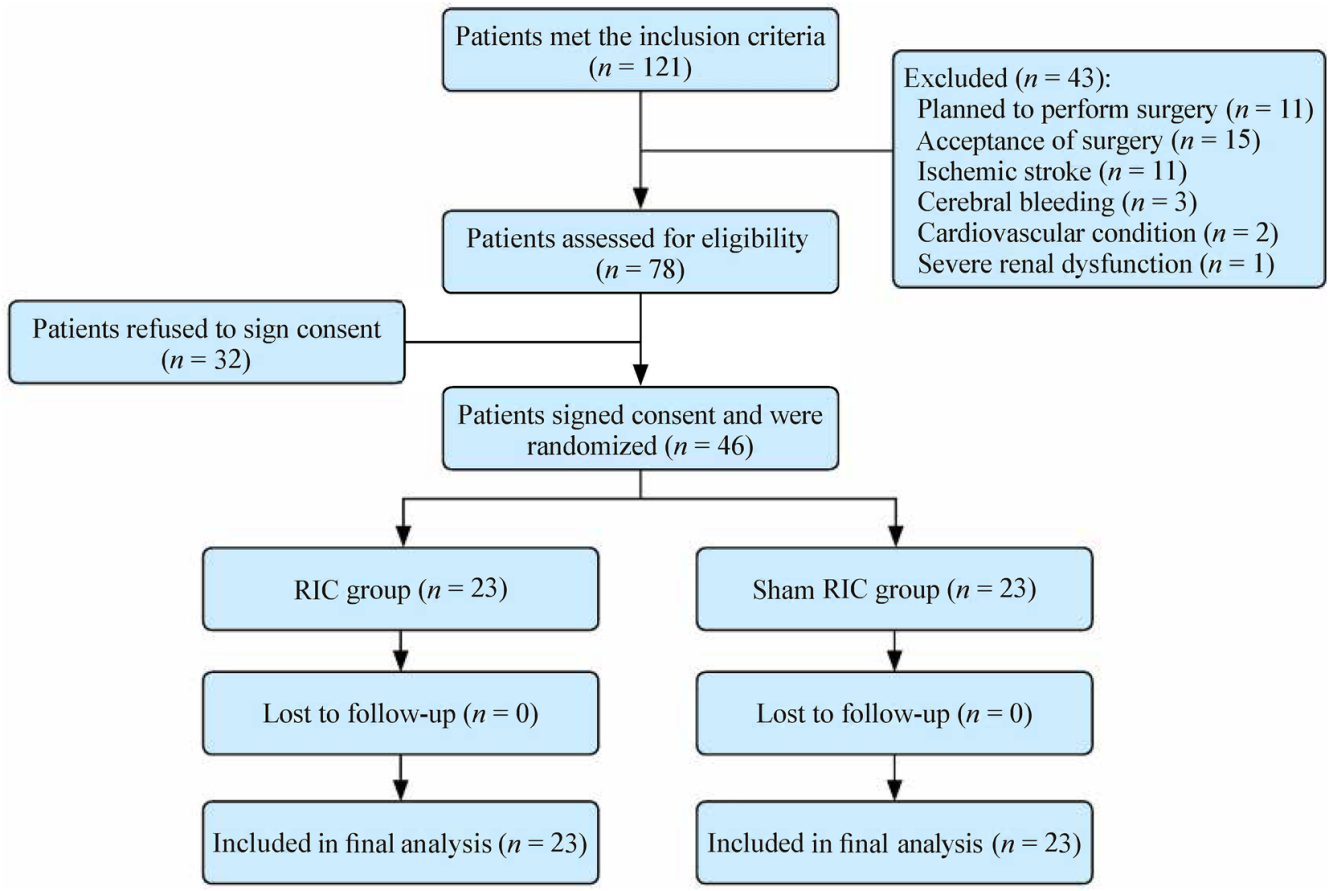
Flow chart of patient selection. *RIC* remote ischemic conditioning

After screening 121 patients diagnosed with MMD, 46 met the inclusion criteria and consented to participate. Twenty-three patients each were allocated to the RIC or control groups. All patients were present at the 1- and 3-year follow-up visits (Fig. 1), and all patients maintained ≥ 80% monthly treatment compliance.

### Baseline characteristics

There were no significant differences in age, sex, family history, stenosis location, posterior circulation involvement, or Suzuki staging between the two groups. The average ages were 7.78 ± 2.76 and 7.69 ± 2.72 years in the sham RIC and RIC groups, respectively (*P* = 0.92). The median Suzuki stage was grade 2, ranging from grade 1 to grade 4 (*P* = 0.79) in both the groups. Two patients had an MMD family history and 13 patients had posterior circulation involvement. The baseline characteristics of the 46 patients are summarized in Table 1.

**Table 1:** Basic demographic data of patients with moyamoya disease

Variables	RIC group (*n* = 23)	Sham RIC group (*n* = 23)	*P*
Female sex, *n* (%)	10 (43.5)	10 (43.5)	1.00
Age (*y*), mean ± SD	7.69 ± 2.72	7.78 ± 2.76	0.92
Family history	2	0	0.49
Location of stenosis			0.75
Unilateral	8	7	
Bilateral	15	16	
Posterior circulation involvement	7	6	0.74
Suzuki stage, median (range)	2 (1–4)	2 (1–4)	0.79

*RIC* remote ischemic conditioning, *SD* standard deviation

### Efficacy outcomes

**Fig. 2:**
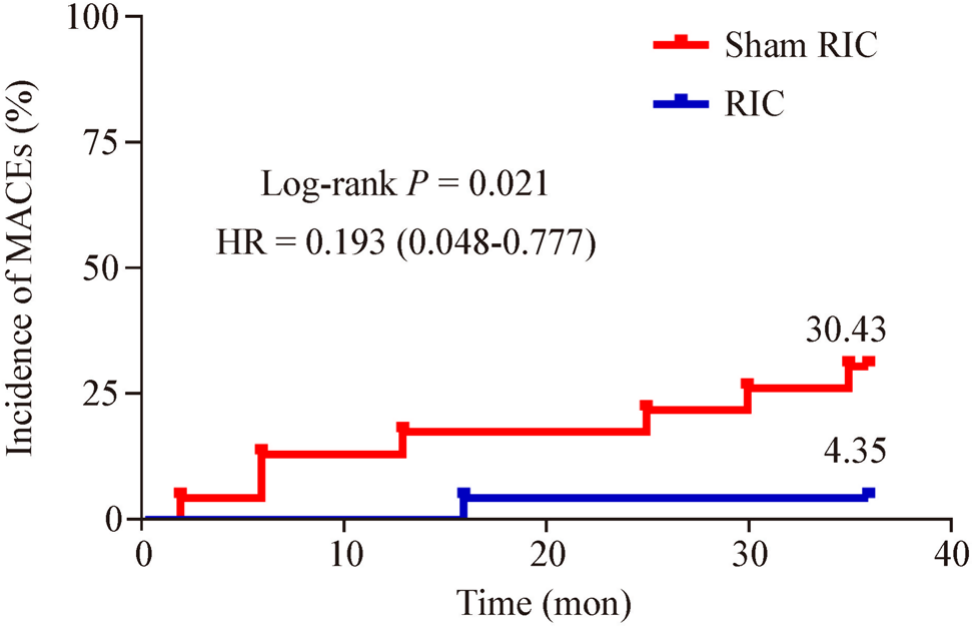
Cumulative incidence of major adverse cerebrovascular events (MACEs) at 36 months. *HR* hazard ratio, *RIC* remote ischemic conditioning

A total of 46 patients completed the 1- and 3-year follow-ups. At the 1-year follow-up, three (6.52%) patients reported MACEs, all of whom were from the sham RIC group. During the 3-year follow-up, eight (17.39%) patients had MACEs, including seven (30.43%) patients from the sham RIC group and one (4.35%) patient from the RIC group. The frequency of MACEs between the RIC and sham RIC groups was analyzed using Kaplan–Meier analysis (Fig. 2), which revealed a significant MACE frequency reduction after RIC [log-rank test (Mantel–Cox), *P* = 0.021].

At the 1-year follow-up, two (4.35%) patients from the sham RIC group had TIAs, and one (2.17%) patient underwent revascularization. At the 3-year follow-up, two (4.35%) patients showed new ischemic lesions on DWI, four (8.70%) experienced TIAs, and two (4.35%) underwent revascularization surgery for a non-cerebrovascular event condition. Only one patient had TIAs in the RIC group, whereas the rest were in the sham RIC group. No cerebral hemorrhagic events were observed during any follow-up period. Table 2 lists the complete data entries.

**Table 2:** Clinical events in patients with MMD treated with RIC or sham RIC

Variables	RIC group (*n* = 23)	Sham RIC group (*n* = 23)
1-y follow-up, *n* (%)		
Incidence of the MACEs	0 (0.00)	3 (13.04)
Incidence of the IS	0 (0.00)	0 (0.00)
Incidence of the TIAs	0 (0.00)	2 (8.70)
Incidence of the revascularization surgery	0 (0.00)	1 (4.35)
3-y follow-up, *n* (%)		
Incidence of the MACEs	1 (4.35)	7 (30.43)
Incidence of the IS	0 (0.00)	2 (8.70)
Incidence of the TIAs	1 (4.35)	3 (13.04)
Incidence of the revascularization surgery	0 (0.00)	2 (8.70)

*MMD* moyamoya disease, *RIC* remote ischemic conditioning, *MACEs* major adverse cerebrovascular events, *IS* ischemic stroke, *TIAs* transient ischemic attacks

**Fig. 3:**
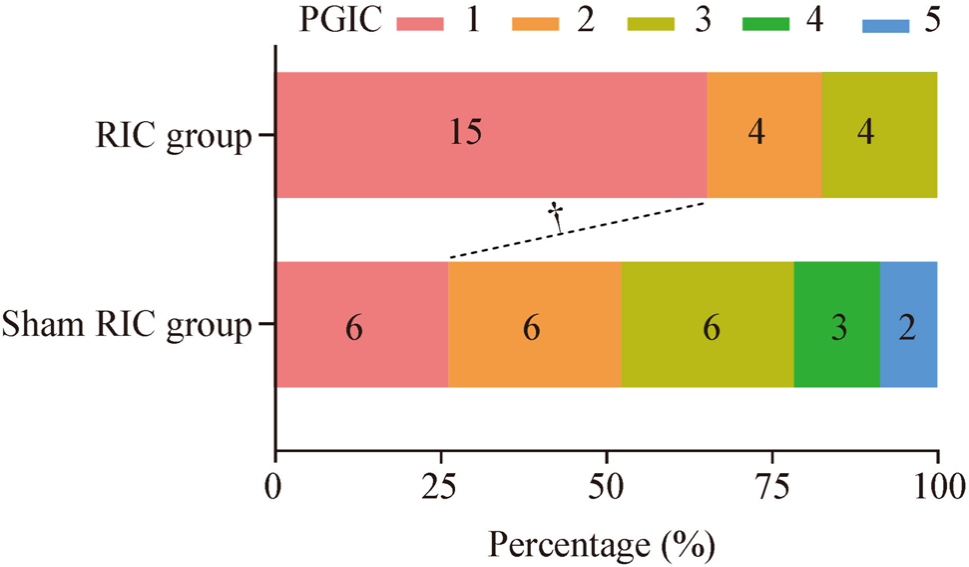
Patient global impression of change scales for patients with moyamoya disease at 1-year follow-up. *RIC* remote ischemic conditioning. ^†^*P* < 0.01

In terms of PGIC, 15 (65.2%) patients in the RIC group reported that their symptoms were significantly relieved or disappeared, four (17.4%) reported that their symptoms were slightly relieved, and four (17.4%) reported that their symptoms remained unchanged during the 1-year follow-up. In the sham RIC group, six (26.1%) patients reported that their symptoms were significantly relieved or disappeared, six (26.1%) reported that their symptoms were slightly relieved, and six (26.1%) reported that their symptoms remained unchanged. Overall, the rate of clinical improvement was higher in the RIC group than in the sham RIC group (65.2% vs. 26.1%, *P* < 0.01). Figure 3 and Table 3 present the complete data entries.

**Table 3:** PGIC scales for patients with MMD at 1-year follow-up

PGIC	RIC group (*n* = 23)	Sham RIC group (*n* = 23)	*P*
Clinical improvement	15 (65.2)	6 (26.1)	< 0.01
Significant reduction or elimination of symptoms	15 (65.2)	6 (26.1)	0.04
Mild attenuation	4 (17.4)	6 (26.1)	
No change	4 (17.4)	6 (26.1)	
Mild deterioration	–	3 (13.0)	
Significant deterioration	–	2 (8.7)	

Data are presented as *n* (%). The PGIC consists of a 5-point scale: 1 = significant reduction or elimination of symptoms; 2 = mild attenuation; 3 = no change; 4 = mild deterioration; 5 = significant deterioration; significant reduction or elimination of symptoms was regarded as clinical improvement. *MMD* moyamoya disease, *RIC* remote ischemic conditioning, *PGIC* Patient Global Impression of Change

### Safety outcomes

Two (8.70%) patients in the RIC group reported skin ecchymosis, whereas none of the patients in the sham RIC group experienced adverse events. The occurrence of unfavorable events did not significantly differ between the two groups (*P* = 0.244). Despite device-related adverse events in the RIC group, no significant discomfort due to RIC treatment was reported. All patients tolerated the RIC strategy and completed the entire study.

### Cerebral hemodynamic results

A total of 19 MCA with MV greater than 159 cm/s at baseline were detected by TCD, with ten vessels in the RIC group and nine vessels in the sham RIC group. There were no statistical differences in PSV, EDV, and MV between the RIC and sham RIC groups at baseline [PSV: 302.5 (IQR = 259.3–357.8) vs. 342.4 (IQR = 272.1–353.1) cm/s, *P* = 0.57; EDV: 178.5 (IQR = 140.5–215.5) vs. 216.0 (IQR = 161.1–231.7) cm/s, *P* = 0.37; MV: 219.8 (IQR = 171.2–263.0) vs. 252.7 (IQR = 193.5–275.7) cm/s, *P* = 0.37]. At the 1-year follow-up, PSV, EDV, and MV in the RIC group were low compared with those in the sham RIC group [PSV: 257.5 (IQR = 79.3–314.3) vs. 302.0 (IQR = 258.7–342.1) cm/s, *P* = 0.22; EDV: 125.5 (IQR = 56.5–183.0) vs. 187.5 (IQR = 135.8–210.0) cm/s, *P* = 0.07; MV: 168.8 (IQR = 64.1–226.8) vs. 226.7 (IQR = 181.3–260.3) cm/s, *P* = 0.08]. In addition, there was a greater median reduction in the PSV, EDV, and MV in the RIC group than in the sham RIC group. Table 4 shows the cerebral hemodynamics in patients with TCD.

**Table 4:** Cerebral hemodynamics evaluation for patients with MMD at 1-year follow-up

Variables	RIC	Sham RIC	*P*
PSV-baseline	302.5 (259.3–357.8)	342.4 (272.1–353.1)	0.57
PSV-follow-up	257.5 (79.3–314.3)	302.0 (258.7–342.1)	0.22
ΔPSV	45 (− 46.3–263.0)	8.2 (− 13.7–38.9)	0.74
ΔPSV%	19.2 (− 16.5–76.9)	2.4 (− 3.9–13.2)	0.68
EDV-baseline	178.5 (140.5–215.5)	216.0 (161.1–231.7)	0.37
EDV-follow-up	125.5 (56.5–183.0)	187.5 (135.8–210.0)	0.07
ΔEDV	34.5 (− 39.8–169.8)	2.4 (− 2.6–37.7)	0.62
ΔEDV%	22.3 (− 29.3–71.1)	1.3 (− 1.5–21.8)	0.62
MV-baseline	219.8 (171.2–263.0)	252.7 (193.5–275.7)	0.37
MV-follow-up	168.8 (64.1–226.8)	226.7 (181.3–260.3)	0.08
ΔMV	38.0 (− 50.1–189.3)	4.0 (− 6.3–38.1)	0.62
ΔMV%	20.8 (− 28.4–73.6)	1.4 (− 2.6–17.8)	0.62

*MMD* moyamoya disease, *RIC* remote ischemic conditioning, *PSV* peak systolic velocity, *ΔPSV* change in the mean peak systolic velocity, *ΔPSV%* percent change in the mean peak systolic velocity, *EDV* end-diastolic velocity, *ΔEDV* change in the mean end-diastolic velocity, ΔEDV% percent change in the mean end-diastolic velocity, *MV* mean velocity, *ΔMV* change in the mean velocity, *ΔMV%* percent change in the mean velocity

## Discussion

Long-term daily RIC is a noninvasive, safe, and well-tolerated treatment for adult patients with MMD [13, 14]. Long-term RIC intervention without reconstructive surgery is safe and feasible in asymptomatic children with MMD. RIC reduced the cumulative incidence of MACEs, and the severity of cerebrovascular ischemic events in the intervention group was mild. Most patients who received RIC treatment reported symptom alleviation. These trials suggest that RIC is highly effective at preventing cerebrovascular ischemic events in children with MMD.

Ischemia is dominant in children with MMD, accounting for 6%–10% of all strokes and TIAs, with a high risk of recurrence [26–28]. One multicenter cohort study of pediatric MMD also showed that cerebrovascular events (ischemia and/or TIA) were the most common clinical manifestations [29]. Epidemiological investigation of MMD in China shows that the most common initial clinical presentation in children is TIAs, accounting for 63.3% of the cases [3]. Furthermore, the natural progression of MMD is much more rapid in pediatric patients than in adults. In children, this disease can result in progressive and permanent functional impairments. Infarction on presentation was associated with an approximately threefold increased risk of unfavorable clinical outcomes in pediatric MMD [30]. Stroke is a major cause of cognitive and emotional disorders that result in learning disabilities, memory loss, and inattention [31]. Therefore, cerebrovascular events are the primary outcomes that must be evaluated.

With the current emphasis on physical examinations and the development of noninvasive diagnostic technologies, such as MRI and magnetic resonance angiography, the incidence of asymptomatic MMD is higher than previously estimated [19]. In adults, hemispheres with asymptomatic MMD may have a 1.0% annual risk of stroke during the first five years [18]. Incidentally discovered asymptomatic moyamoya in children has the potential for radiographic and clinical progression [32]. Radiographic progression occurs in most asymptomatic pediatric patients, manifesting as increased vessel narrowing, slow cerebral blood flow, and signs of radiation infarction, often leading to subsequent clinical manifestations of cerebral ischemia [32, 33]. Children with moyamoya arteriopathy, even without ischemic symptoms, are at a risk of stroke, and an unfavorable clinical prognosis remains possible [33].

Specific criteria for determining the timing and nature (unilateral or bilateral) of surgery in children with asymptomatic MMD have not been well established. As such, immediate surgery may not always be necessary [34]. Instead, individualized neuroimaging assessments should be performed. In this study, two patients in the control group underwent EDAS surgery during clinical follow-up because of rapid imaging progression and worsening of nonspecific symptoms (headache, dizziness, or insomnia). Further prospective studies are necessary to optimize management guidelines for asymptomatic pediatric patients [29, 32, 34].

RIC is a therapeutic strategy used to protect tissues and organs from the harmful effects of ischemia/reperfusion injury. RIC has broad applications in the treatment of cerebrovascular diseases [35]. The safety of RIC has been demonstrated in adult patients with acute ischemic stroke and aneurysmal subarachnoid hemorrhage, and RIC has potential protective effects [36, 37]. Long-term RIC has been increasingly investigated as a means of ameliorating injury following chronic ischemic insults to the brain, such as intracranial atherosclerotic stenosis (ICAS) [38] and cerebral small vessel disease (CSVD) [39]. A multicenter randomized controlled trial confirmed that long-term RIC lowers the risk of cardiovascular and cerebrovascular events in patients with ischemic stroke or TIA caused by symptomatic ICAS [38]. A small pilot study on 17 patients with CSVD found that RIC was effective at slowing cognitive decline and reducing white matter hyperintensities [39]. RIC also increases cerebral blood flow in patients with MMD, reducing the risk of ischemic stroke recurrence and the incidence of TIA [13, 14]. The results of this study confirmed that twice-daily RIC treatment for asymptomatic children with MMD over one year could effectively prevent the occurrence of ischemic cerebrovascular events. Patients in the RIC group tended to have a lower incidence of MACEs, and the cumulative incidence of MACEs after three years of follow-up was significantly lower. The participants in the present study were mainly asymptomatic, without obvious hemodynamic changes, and the incidence of cerebrovascular events was relatively low.

A randomized controlled trial involving adult patients with MMD treated with RIC twice daily for one year showed that the incidence of MACEs in the RIC group was lower than that in the control group, although the difference was not statistically significant [14]. A cohort study with an average follow-up of 8 years investigated chronic RIC for symptomatic internal carotid artery or middle cerebral artery occlusion, showing promising results for the cumulative incidence of cerebrovascular disease [40]. Based on previous research on RIC treatment for cerebrovascular diseases, along with the clinical features of asymptomatic children with MMD in this study, and considering the feasibility of the study, we initiated a one-year intervention plan with twice-daily sessions. Under conditions of good patient compliance and no serious adverse events, the present study was conducted for three years of follow-up, and favorable outcomes were observed.

The mechanism underlying the protective effects of RIC against MMD remains unclear and requires further investigation. Currently, the potential mechanisms of RIC include neuroprotection through an anti-inflammatory response, neuronal safeguarding against excitotoxicity, mitochondrial protection, activation of circulating inflammasomes and/or transcriptional regulation of neuroprotective pathways, and improvement of collateral circulation [41–43]. RIC could alleviate hemodynamic instability in adult patients with ischemic MMD, as demonstrated by the significant decrease in PSV on TCD after RIC treatment [13]. We also evaluated the changes in cerebral artery blood flow velocity, finding that PSV, EDV, and MV decreased more significantly in the RIC group after one year of intervention. This suggests that RIC alleviates hemodynamic instability. A recent cohort study examined cerebral blood flow velocity using TCD to assess cerebral hemodynamic stability in children with MMD [23]. However, the current study did not demonstrate a statistical variance among TCD-related indicators, which could be, in part, due to a combination of factors such as inherently milder arteriopathy, earlier implementation of therapies in the disease course, a limited sample size, and brief intervention and follow-up periods.

Patients’ subjective feelings are an important aspect of disease assessment. During the progression of asymptomatic MMD, individuals typically experience nonspecific symptoms such as headaches, dizziness, or insomnia [6]. Most individuals who underwent RIC treatment reported improvement in their symptoms as measured using their PGIC scales. In this study, the PGIC scales reflecting emotional and patient-perceived general health statuses were used as indicators of symptom improvement [13].

This study has several limitations. Firstly, this single-center, small-sample pilot experiment may not fully represent the whole target population, thus the results should be interpreted with caution. Future research should focus on conducting large-sample, multicenter trials to improve the generalizability of the findings. Secondly, while this study assessed clinical events and cerebral hemodynamics using TCD, comprehensive follow-up imaging examinations and body fluid sample collection were not performed to investigate the specific mechanism of RIC in MMD in children. Further exploration into the mechanism of RIC action in MMD is warranted. Thirdly, this study was confined to the age range of 4–14 years. Future studies should include a wider age range to better understand the safety and effectiveness of treating pediatric MMD with RIC. Finally, the follow-up interval was relatively short, which may have limited the long-term findings of the study; longer follow-up intervals may have resulted in more robust findings.

In conclusion, RIC may be a promising noninvasive method for managing pediatric MMD. For asymptomatic pediatric MMD, long-term RIC is well tolerated and may lower the incidence of ischemic cerebrovascular episodes and prolong the waiting time for surgery.

## Data Availability

The data of this study are available on reasonable request to the corresponding author.
